# Prevalence and molecular characterisation of *Giardia* spp. and *Cryptosporidium* spp. at the wildlife-livestock interface in Laikipia, Kenya

**DOI:** 10.1016/j.ijppaw.2026.101263

**Published:** 2026-07-20

**Authors:** Andrew M. Halls, Alice M. Burton, Olatunji Johnson, Catherine Walton, Charis Enns, Kathryn J. Else, Ian Hall, Melanie Wangui, Cianjoka Gichuyia, Eric M. Fèvre, Susanne Shultz

**Affiliations:** a Department of Earth and Environmental Science, The University of Manchester, Manchester, UK; b Department of Mathematics, University of Manchester, Manchester, UK; c Global Development Institute, The University of Manchester, Manchester, UK; d Lydia Becker Institute, The University of Manchester, Manchester, UK; e Christabel Pankhurst Institute, University of Manchester, Manchester, UK; f Losiaba Conservancy, Laikipia, Kenya; g Institute of Infection, Veterinary, and Ecological Sciences, University of Liverpool, Liverpool, UK; h International Livestock Research Institute (ILRI), Nairobi, Kenya

**Keywords:** Giardia, Cryptosporidium, Wildlife-livestock interface, Zoonoses, Wildlife, Livestock, Kenya, East Africa

## Abstract

Giardia and Cryptosporidium are important causes of diarrhoeal disease in humans, particularly in sub-Saharan Africa, with several species and genotypes capable of zoonotic transmission. Central Laikipia, Kenya, represents a shared landscape characterised by close human-livestock-wildlife interactions. Traditional pastoral systems and commercial ranching coexist alongside high wildlife diversity offering high potential for interspecies disease transmission. Despite this, molecular data on protozoan parasites in animal hosts remains limited across the region. We collected faecal samples opportunistically from six wildlife and four livestock species over three years in Laikipia and screened them using molecular methods to investigate the sample prevalence and genetic diversity of *Giardia* and *Cryptosporidium*. *Giardia* occurrence was consistently high, ranging from 21% in in samples from 2022 to 27% in samples from 2025, whereas *Cryptosporidium* occurrence remained low (0–5%) over the same period. Multilocus sequencing was partially successful for a subset of samples at the *beta-giardin*, *triosephosphate isomerase*, and *glutamate dehydrogenase* genes. This identified predominantly livestock-adapted *Giardia duodenalis* assemblage E, with much lower occurrence of potentially zoonotic assemblages AI and AII in livestock. Sequencing of the *18S SSU* gene identified multiple *Cryptosporidium* species These were mostly ungulate specific species but did include the potentially zoonotic *C. ubiquitum*. The detection of both ungulate-specific and possibly zoonotic genotypes highlights possible One Health implications within this shared landscape. While potentially zoonotic genotypes indicate there may be public health risk through animal-to-human transmission, the predominance of ungulate-specific genotypes suggests higher potential for animal-to-animal transmission cycles, with potential indirect effects on human health through impacts on livestock productivity and growth.

## Highlights

• High prevalence of *Giardia* and low prevalence of *Cryptosporidium* found.
• Multiple assemblages of *G. duodenalis* and species of *Cryptosporidium* found.
• First report of *Giardia* in buffalo, Plains zebra, African elephant and impala.
• First report of *G. duodenalis* assemblage A in camel, impala and Plains zebra.
• Animal adapted species and assemblages found more than zoonotic ones.

## Graphical abstract

**Figure:**
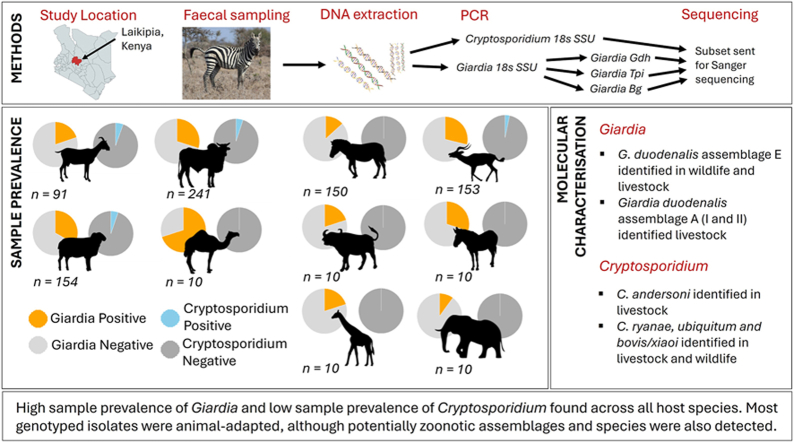

## 1 Introduction

Transhumance pastoralism is the dominant livelihood in East Africa's rangelands. Historical and contemporary issues in land ownership have resulted in the landscape changing, however large numbers of people still practice pastoralism despite restrictions to movement and limited land availability. This has led to close and frequent interactions between people, their livestock and wildlife across the landscape (Homewood et al., 2004). These interactions are particularly intense during periods of stress such as droughts where resource availability, especially water, is limited. This intense human-livestock-wildlife interface is a potential hotspot for disease transmission.

Laikipia County is located in central Kenya. The study region in central Laikipia is an area where privately owned ranches, private conservation-focused areas and pastoralist communities all exist (Gadd, 2005). The predominant habitat in Laikipia is wooded savannah, which makes it suboptimal for agriculture but suitable for livestock production (Gadd, 2005). Despite the absence of designated national parks, Laikipia is home to a significant amount of Kenyan wildlife. The region features an open landscape with limited barriers between properties. This allows relatively unrestricted wildlife movement across the region (Mworia, 2006). Livestock movements are generally constrained, with herders ensuring animals typically stay within defined property areas. Such shared use of space and resources across this landscape creates opportunities for the transmission of gastrointestinal parasites between wildlife, livestock, and people.

Diarrhoea is in the top five causes of mortality in under-fives globally, and children in Sub-Saharan Africa (SSA) are up to 14 times more likely to die before the age of five than in Europe or North America (World Health, 2022). East Africa has the highest diarrhoeal incidences in children under 5 in SSA (Thystrup et al., 2024). Both *Giardia* and *Cryptosporidium* are genera of protozoan parasites that are species diverse and infect most if not all vertebrates globally. Both these pathogens are transmitted via faecal-oral routes. Hosts infected by the parasite shed viable *Giardia* cysts or *Cryptosporidium* oocysts that, once swallowed by a susceptible host, can initiate infection (Maxamhud et al., 2025). These infectious cysts and oocysts can also be transmitted through contaminated water (Hunter and Thompson, 2005).

As multi-host parasite genera with both broad zoonotic and host-specific species, *Giardia* and *Cryptosporidium* represent important One Health concerns at human–livestock–wildlife interfaces. Although not all species or genotypes of these parasites are zoonotic, *Giardia* has been identified as the second leading cause of diarrhoea cases in under five year olds in SSA after *E. coli* (Thystrup et al., 2024). Similarly, *Cryptosporidium* is also responsible for a substantial proportion of cases across the region (Hossain et al., 2023). There are 6 widely accepted species of *Giardia* with *G*. *duodenalis* being the most significant from a zoonotic perspective. Eight distinct genotypes or assemblages (A to H) of *G. duodenalis* have been identified. Assemblages A and B have the broadest host ranges, including humans, and are considered to have the highest zoonotic impact (Heyworth, 2016). Within the genus *Cryptosporidium*, at least 44 species have been identified. Among these species, 19 have been reported to infect humans, with the most prominent ones being *C. hominis* and *C. parvum* (Ryan et al., 2021). *C. parvum* is considered the most important zoonotic species for human infections, and is found in a wide range of hosts, including humans, wild and domestic ungulates, carnivores, rodents, and fish (Xiao et al., 1999b).

Whilst there are studies that evaluate prevalence of *Giardia* and *Cryptosporidium* in East Africa these predominately focus on human cases (Mbae et al., 2016), and to a lesser extent livestock (Kanyari et al., 2010). In contrast, there is very limited knowledge of prevalence in wildlife within these pastoralist landscapes. Here we collected and screened 343 faecal samples from 6 wildlife species and 496 faecal samples from 4 livestock species across central Laikipia for both *Giardia* and *Cryptosporidium*. The objectives of this study were to calculate the observed prevalence of both parasites in the samples collected from sympatric wildlife and livestock populations in Laikipia County, Kenya, and to characterise the present species and assemblages. To our knowledge this is the first study to consider these parasites in East African wildlife in recent times.

## 2 Methods

### 2.1 Study regions

We selected four neighbouring properties in Laikipia for this study. Mpala and Loisaba are privately owned properties. Mpala is a research centre which prioritises research and conservation whilst maintaining small cattle, camel and sheep herds. Loisaba is a wildlife conservancy whose main industry focuses on sustainable tourism and conservation; however, it still maintains a commercial ranching operation with cattle, sheep, goats and camel. Both these properties have planned grazing and are home to large densities and diversity of wildlife (Gitau et al., 2025; Walker et al., 2014). Il Motiok and Koija are two community lands within Naibung'a Lower Conservancy (Lalampaa et al., 2016). These are home to pastoralist Maasai communities who keep herds of cattle, sheep, goats and camels. These areas have higher densities of livestock (Gitau et al., 2025), as a result of this, alongside increased human activities we observed much lower densities and diversity of wildlife however anecdotal evidence suggests that they use these areas more frequently at night when there is less human activity.

### 2.2 Sample collection

**Fig. 1:**
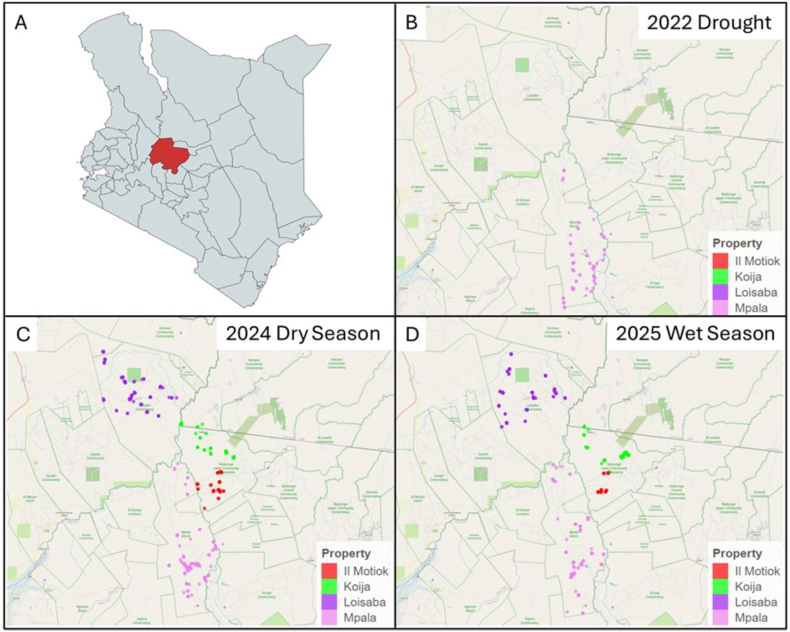
Maps of the study sites and sample distributions. A - Map showing the location of Laikipia in Kenya (shaded in red). B-D Maps showing samples collected in 2022 (B), 2024 (C) and 2025 (D) respectively. 2022 was a drought year, 2024 was a dry season and 2025 was a wet season. Dots indicate individual samples collected and are coloured by the property; Il Motiok in red, Koija in green, Loisaba in purple, and Mpala in pink.

We collected faecal samples opportunistically from five target species: Plains zebra (*Equus quagga*), impala (*Aepyceros melampus*), cattle *(Bos indicus*), sheep (*Ovis aries*) and goats (*Capra aegagrus hircus*). It was not always possible to collect samples from all target species at every sampling time point on each property, either due to changes in livestock stocks or to a lack of wildlife observations. We collected samples on Mpala during March–April 2022 and across all four properties between February–March 2024 and April–June 2025 (Fig. 1). The sampling period in 2022 was during a drought in the region, 2024 was during the dry season and 2025 during the wet season. Samples from additional species were collected opportunistically.

We implemented an opportunistic sampling plan. We aimed to spatially distribute collections across each property; sampling locations were constrained by animal detectability and road access. We targeted different areas of each property for collections on different days, the numbers of wildlife on the properties on which samples were collected are high meaning the chances of repeat sampling were slim. Repeat sampling of livestock was avoided by not sampling a herd more than once. Due to the opportunistic nature of sampling prevalence is based on observed occurrence within our samples rather than wider population level estimates.

Livestock samples were collected with the owner's consent by observing defecation and collecting. We observed wild animals defecating from a safe distance and collected samples once the animals had moved away to minimise disturbance. We collected all samples in sterile plastic bags avoiding collecting faeces in contact with the floor to avoid environmental contamination. We transported samples in cooler boxes with ice packs and stored them at −20 °C at Mpala Research Centre (MRC) prior to DNA extraction and screening at MRC.

### 2.3 DNA extraction

We extracted DNA from faecal samples using Zymo Quick-DNA Fecal/Soil Microbe Miniprep Kit (D6010, Zymo Research, Irvine, CA, USA) following the manufacturer's instructions. For disruption/cell lysis, we included a bead beating step using a Benchmark bead bug 6/bead blaster 24 (Benchmark Scientific, Sayreville, NJ, USA) for 4 cycles of 50 s at 7 m/s for a total of 200 s of bead beating.

### 2.4 Amplification of *Giardia* and *Cryptosporidium* 18S SSU genes

We performed PCRs using GoTaq G2 Hotstart Taq Polymerase (M7405, Promega, Madison, WI, USA) in 25 μl reactions consisting of 1x Gotaq G2 hotstart buffer, 2.5 mM MgCl_2_, 0.2 mM of each dNTP, 0.6 μM of each forward and reverse primer, and 0.625 U Taq polymerase. For the primary step PCRs, we added 1 μl of extracted DNA, and for the nested step we added 1 μl of product from the primary step. Each reaction included positive and negative controls. We ran positive controls in the same reaction mixtures as the samples, but using reference DNA from either *G. duodenalis* or *Cryptosporidium spp*. instead of sample DNA. These were provided by the Welsh Cryptosporidium Reference Unit. The negative controls consisted of the same reaction mixture as the rest of the samples minus the sample DNA.

We detected *Giardia* spp. using a nested PCR targeting the 18S SSU gene (ElBakri et al., 2014). We amplified the 18S SSU region of *Cryptosporidium* using the method described by by (Xiao et al., 1999a, 1999b). Primers and reaction conditions can be found in Table A.1 and Table A.2 respectively.

### 2.5 Amplification of *Giardia beta-giardin, triosephosphate isomerase and glutamate dehydrogenase* genes

Whilst the 18S SSU can be used for the species identification of *Cryptosporidium*, in *Giardia* its highly conserved nature across species/assemblages limits its reliability for species identification (Caccio et al., 2008). Therefore, we used a common multilocus approach targeting the beta-giardin (*Bg*) (Kuthyar et al., 2021), triosephosphate isomerase (*Tpi*) (Sulaiman et al., 2003), and glutamate dehydrogenase (*Gdh*) (Read et al., 2004) genes to effectively identify species and assemblages (Zheng et al., 2014). We selected a subset of 96 samples for *Giardia* sequencing PCRs to represent a range of host species and sampling years. Of these 33 successfully amplified at the *Bg* gene, 13 at the *Tpi* and 26 at the *Gdh.* We used the same reaction mixtures as for the 18S SSU PCRs. Primers and reaction conditions can be found in Table A.1 and Table A.2 respectively.

### 2.6 PCR product visualisation, purification and sequencing

After PCR amplification, we visualised products on a 1.5% agarose gel stained with safeview classic (G108, Applied Biological Materials Inc., Richmond, BC, Canada) to identify positives. We cleaned a selected subset of positive samples using either the exonuclease 1 and FastAP Thermosensitive Alkaline Phosphatase (Thermo Fisher Scientific, Waltham, MA, USA) method following the manufacturer's instructions or using Monarch PCR & DNA Cleanup Kits (T1030S, New England Biolabs, Ipswich, MA, USA) as per manufacturer's instructions.

Successful amplification and clear bands post cleaning were sent for Sanger sequencing in the forward direction at the SEGOLIP unit at the International Livestock Research Institute in Nairobi, Kenya. All sequences were submitted to GenBank under the following accession numbers: Cryptosporidium 18S SSU: PX970961-PX970982, Giardia *Bg*: PX986065-PX986077, *Tpi*: PX986093-PX986100 and *Gdh*: PX986078-PX986092.

### 2.7 Statistical analysis

We conducted all statistical analyses in R v4.5.1 (R Core Team, 2025). Prevalence in this study is defined as proportion of positive occurrences in the collected samples. We quantified uncertainty using 95% Jeffreys confidence intervals for binomial proportions. We restricted further analyses to five focal host species (cattle, sheep, goats, plains zebra, and impala) for which adequate sample sizes were available. Protozoan infection status (1 = positive, 0 = negative) was modelled using generalised linear models (GLMs) with a binomial error distribution and logit link. We fitted separate models for *Giardia* and *Cryptosporidium* infection to evaluate the effects of host species and sampling year. A third, combined model included parasite identity (*Giardia* vs *Cryptosporidium*) as an additional predictor to compare overall infection likelihoods across hosts and sampling periods. For *Cryptosporidium*, sparse infections and zero cell counts in some host species resulted in complete separation; therefore, we fitted bias-reduced logistic regression models using the brglm2 package (Kosmidis, 2025) to obtain finite and stable parameter estimates. We evaluated model significance using Type III likelihood-ratio χ^2^ tests (carAnova) (Fox and Weisberg, 2019), and model-adjusted probabilities with Tukey-adjusted pairwise contrasts were obtained from estimated marginal means (emmeans, type = “response”) (Lenth, 2025). We performed sensitivity analysis using binomial generalised mixed models with year included as a random intercept (Table A6). Coinfections between *Giardia* and *Cryptosporidium* were assessed using Fisher's exact tests. We produced maps using mapchart.net and in R using the leaflet package (Cheng et al., 2025).

### 2.8 Sequence processing and phylogenetic analysis

We quality-trimmed raw sequences in UGENE v46.0 (Okonechnikov et al., 2012) by removing low-quality bases and trimming read ends. We aligned sequences by locus with reference sequences representing known species and assemblages from NCBI GenBank (Clark et al., 2016) using the MUSCLE algorithm in MEGA X (Kumar et al., 2018) with default parameters. We trimmed alignments to uniform length, and exported them as FASTA files for analysis in R.

We imported sequence alignments with Biostrings (Pagès et al., 2025) and processed them in ape (Paradis and Schliep, 2019), DECIPHER (Wright, 2016), and phangorn (Schliep, 2010; Schliep et al., 2017). For the *Cryptosporidium* 18S locus, alignment columns containing more than 40% gaps were excluded to reduce alignment noise; no additional column masking was applied to protein-coding loci (*Bg, Tpi, Gdh*). We estimated neighbour-joining starting trees from model-based distances (*dist.ml).* We then fitted maximum-likelihood trees under alternative substitution models (JC, HKY, GTR ± G ± I) using *pml* and *optim.pml*, and selected the best model based on the lowest corrected Akaike Information Criterion (AICc). We assessed node support by 1000 non-parametric bootstrap replicates (*bootstrap.pml*) and generated a bootstrap consensus tree by collapsing nodes with <70% support (*di2multi*). We visualised final trees in ape and exported them in Newick format. We visualised and annotated the final trees using the Interactive Tree of Life version 7 online platform (Letunic and Bork, 2024). We assigned *Cryptosporidium* species and *Giardia duodenalis* assemblages based on clustering with reference sequences.

### 2.9 Ethical approval and permits

All sampling conducted in this study was non-invasive and involved the collection of freshly deposited faecal material only, with no handling or disturbance of animals. Wildlife faecal samples were collected opportunistically following observation of defecation from a safe distance. Livestock sampling was conducted with the approval of the Directorate of Veterinary Services (approval reference: MOALF/SDL/DVS/DS/RESEARCH/VOL 11/119) and permission of animal owners and herd managers.

Ethical approval for the study was granted by the University of Manchester Research Ethics Committee under Category D (approvals D097 and D068). Research authorisation to conduct research in Kenya was obtained from the National Commission for Science, Technology and Innovation (NACOSTI) under permits NACOSTI/P/22/15559, NACOSTI/P/23/31905, and NACOSTI/P/25/4173899. Permission to conduct wildlife-related research was also granted by the Wildlife Research and Training Institute (WRTI) under permits WRTI-0152-02-22, WRTI-0393-01-24, and WRTI/RPC/2025-307313. Permission to access and sample on private and community group ranches was obtained from property managers and community representatives prior to fieldwork.

All research was conducted in accordance with institutional ethical guidelines and Kenyan national regulations governing wildlife and livestock research.

## 3 Results

We collected a total of 839 faecal samples from 10 species in Laikipia across three years (Table 1).

**Table 1:** Number of samples analysed per sampling location/period per species.

Species	2022	2024	2025
Buffalo (*Syncerus caffer*)	0	10	0
Camel (*Camelus dromedarius*)	0	10	0
Cattle (*Bos indicus*)	28	123	90
Elephant (*Loxodonta africana*)	0	10	0
Reticulated giraffe (*Giraffa reticulata*)	0	10	0
Goat (*Capra hircus*)	0	46	45
Grevy's zebra (*Equus grevyi*)	0	10	0
Impala (*Aepyceros melampus*)	28	64	61
Plains zebra (*Equus quagga*)	29	59	62
Sheep (*Ovis aries*)	0	79	75

### 3.1 Overall sample prevalence

**Fig. 2:**
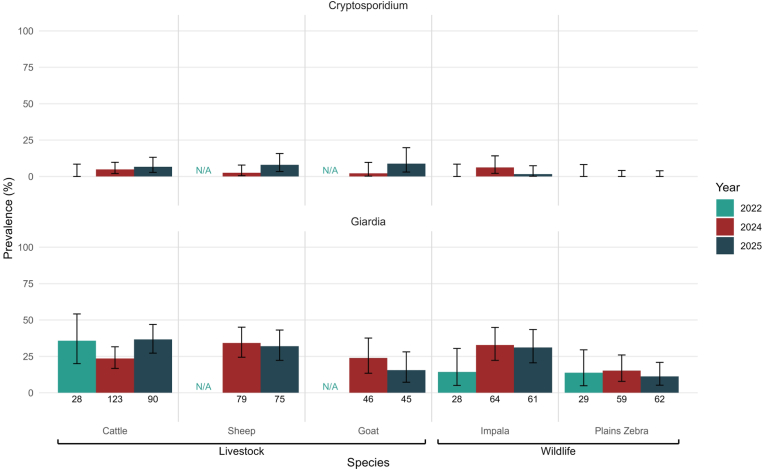
Sample prevalence of *Giardia* and *Cryptosporidium* of the 5 target ungulate species across years. Bar plots show the observed sample prevalence (%) of *Cryptosporidium* (top) and *Giardia* (bottom) in domestic and wild ungulates sampled in Laikipia (2022, 2024, 2025). Each colour represents a sampling year; 2022 is in light green, 2024 in mustard, and 2025 in dark green. Numbers below bars indicate the total number of individuals sampled; “N/A” indicates that a species was not sampled in that dataset, rather than a zero occurrence. Prevalences are proportions of positive samples out of all samples tested. Error bars indicate 95% Jeffreys confidence intervals.

Observed prevalence of gastrointestinal protozoa varied across years, and host species (Fig. 2; Table 2, Table 3). *Giardia* was consistently more prevalent than *Cryptosporidium* in samples across all years and species. Mean *Giardia* sample prevalence across species ranged from 21% in Laikipia 2022 to 27% in Laikipia 2025, whereas mean *Cryptosporidium* infection was consistently low, ranging from 0% in Laikipia 2022 to 5% in 2025. Sample sizes varied between datasets (85–421 samples), but the relative pattern of higher *Giardia* occurrence compared to *Cryptosporidium* was consistent across all years.

**Table 2:** Pooled mean sample prevalence of Giardia and Cryptosporidium across Years. Mean sample prevalence (%, ±standard error (SE)) of Giardia and Cryptosporidium infections in wild and domestic ungulates sampled in Laikipia (2022, 2024, 2025). Sample prevalences shown are pooled across host species within each year and are pooled across each of the properties sampled. The total number of individual faecal samples analysed per dataset is shown.

		*Giardia*		*Cryptosporidium*	
Year	Total samples	Mean Prevalence (%)	SE	Mean Prevalence (%)	SE
Laikipia 2022	85	21.12	4.43	0.00	0.00
Laikipia 2024	421	26.60	2.15	3.09	0.84
Laikipia 2025	333	27.02	2.43	5.04	1.21

**Table 3:** Sample prevalence of Giardia and Cryptosporidium by species. Mean sample prevalence of Giardia and Cryptosporidium infections in Laikipia (2022, 2024, 2025). Sample prevalences shown are pooled across the properties sampled. The total number of individual faecal samples analysed per species is shown 95% Jeffreys confidence intervals are shown.

		*Giardia*			*Cryptosporidium*		
Species	No. of Samples	No. of positive	Prevalence (%)	95% CI	No. of positive	Prevalence (%)	95% CI
Camel (*Camelus dromedarius*)	10	7	70.00	39.57–91.20	0	0.00	0.00-21.72
Cattle (*Bos indicus*)	241	72	29.88	24.36-35.87	12	4.98	2.75-8.27
Goat (*Capra hircus*)	91	18	19.78	12.61-28.83	5	5.49	2.13-11.62
Sheep (*Ovis aries*)	154	51	33.12	26.05-40.81	8	5.19	2.49-9.56
Buffalo (*Syncerus caffer*)	10	2	20.00	4.41-50.28	0	0.00	0.00-21.72
Elephant (*Loxodonta africana*)	10	1	10.00	0.58–33.63	0	0.00	0.00-21.72
Reticulated Giraffe (*Giraffa reticulata*)	10	2	20.00	4.41-50.28	0	0.00	0.00-21.72
Grevy's Zebra (*Equus grevyi*)	10	3	30.00	9.27-60.58	0	0.00	0.00-21.72
Impala (*Aepyceros melampus*)	153	44	28.76	22.03-36.28	5	3.27	1.26-7.01
Plains Zebra (*Equus quagga*)	150	20	13.33	8.61-19.46	0	0.00	0.00-1.66

We evaluated spatial and temporal differences for the 5 target species with the highest sample sizes: cattle, sheep, goats, impala and Plains zebra. Information on the sample prevalence of all species across datasets is displayed in Fig. A1.

*Giardia* infection likelihood differed significantly among host species but not among sampling years (Table 4). Predicted sample infection probabilities were highest in sheep (31.75%, 95% CI: 24.02–40.62%) and cattle (29.11%, 95% CI: 23.23–35.77%), and lowest in Plains zebra (12.99%, 95% CI: 8.46–19.44%) (Table A4). Plains zebra had significantly lower odds of *Giardia* infection than cattle, sheep, and impala (Tukey-adjusted p < 0.01), whereas other interspecific differences were not significant (Table A5).

**Table 4:** Results of generalised linear models (GLMs; binomial error, logit link) testing the influence of host species and sampling year on Giardia and Cryptosporidium infection likelihood, and a combined model including both parasites. Test statistics are likelihood-ratio χ^2^ values from Type III analysis-of-deviance tables. Significant effects (p < 0.05) are shown in bold. Full model coefficients displayed in Table A3.

Model	Predictor	χ^2^	df	*p*-value
*Giardia*	Year	0.61	2	0.74
	**Species**	**21.93**	4	**<0.001**
*Cryptosporidium*	Year	6.36	2	0.06
	**Species**	**11.14**	4	**0.03**
Combined (both parasites)	**Parasite**	**171.90**	1	**<0.001**
	**Species**	**28.07**	4	**<0.001**
	Year	2.29	2	0.3

In contrast, *Cryptosporidium* infections were rare but still showed a significant effect of host species, with no effect of year (Table 4). Estimated sample infection probabilities were uniformly low, ranging from 0.22% in Plains zebra (95% CI: 0.01–3.68%) to 3.39% in cattle (95% CI: 1.25–8.88%) (Table A4). Plains zebra showed lower infection odds in the bias-reduced model (p = 0.05; Table A3) and had the lowest estimated sample infection probability based on marginal means (Table A4); however, pairwise species comparisons were not significant after adjustment for multiple testing (Table A5).

A combined model including both protozoan parasites confirmed strong effects of parasite species and host species on sample infection likelihood, but no influence of year (Table 4). *Giardia* infection was substantially more likely than *Cryptosporidium* infection (odds ratio = 2.22, p < 0.001; Table A5), with mean infection probabilities of 22.65% (95% CI: 18.97–26.82%) and 3.09% (95% CI: 2.09–4.55%), respectively (Table A4). Consistent with the parasite-specific models, Plains zebra had the lowest estimated sample infection probability among host species (Table A4). Pairwise contrasts indicated significantly lower infection odds in Plains zebra relative to cattle, sheep, and impala (Tukey-adjusted p < 0.01; Table A5).

### 3.2 Coinfections

**Fig. 3:**
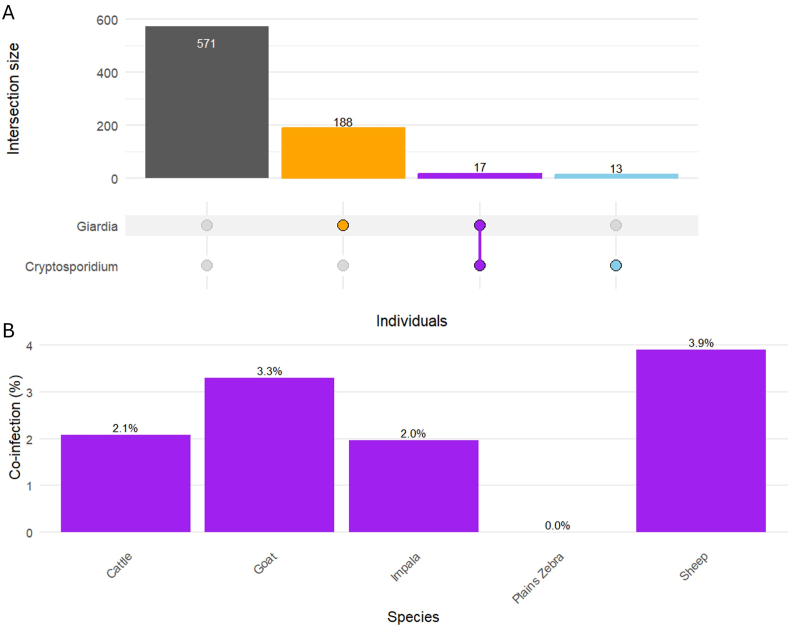
A - Coinfection status of samples. A – Upset plots showing coinfection status of samples, showing the proportion infected with *Giardia* only (orange), *Cryptosporidium* only (blue), both parasites (coinfected, purple), or neither (uninfected, grey). Bars show the intersection size, i.e. the number of hosts with each exact combination of infections. B - Proportion of samples co-infected with both *Giardia* and *Cryptosporidium* across the five target species. Bars show the percentage of sampled animals with simultaneous infection by both parasites. Labels above bars indicate exact sample prevalence values.

The observed number of coinfected samples (n = 17) (Fig. 3A) was substantially higher than expected under the assumption of independent infections (7.79) (χ^2^ = 13.65, *p* = 0.0002), indicating a significant positive association between *Giardia* and *Cryptosporidium* infections. Co-infection occurrence varied across the five focal host species, although overall rates were low (Fig. 3B). Despite these differences in observed proportions no significant variation in co-infection occurrence among species was found (Fisher's exact test, *p* = 0.1167).

### 3.3 Molecular characterisation

**Fig. 4:**
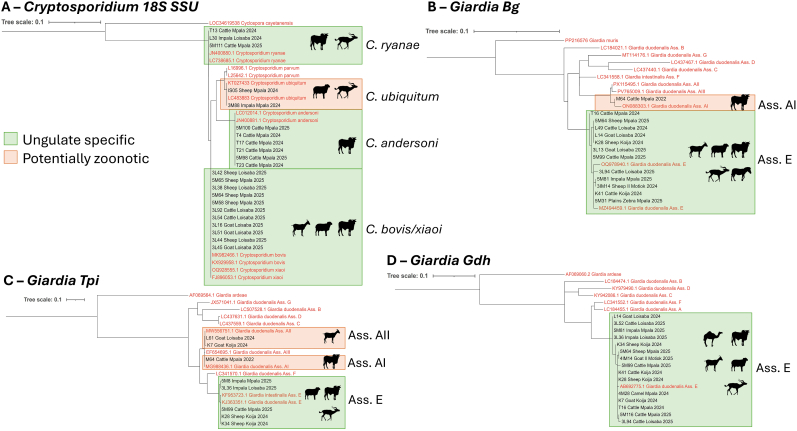
Maximum-likelihood phylogenetic trees used for species and assemblage identification of *Giardia duodenalis* and *Cryptosporidium* isolates from livestock and wildlife in Kenya, based on four loci: (A) *Cryptosporidium spp.* (HKY model), (B) *β-giardin* (*Bg*, GTR + G model), (C) *triose phosphate isomerase* (*Tpi*, GTR + G model), and (D) *glutamate dehydrogenase* (*Gdh*, GTR + G model). Trees were reconstructed in R using the phangorn package, with the best-fitting substitution model selected for each locus based on the lowest corrected Akaike Information Criterion (AICc). Node support was assessed using 1000 non-parametric bootstrap replicates, and nodes with bootstrap support <70% were collapsed in the final consensus trees. Study sequences were assigned to species or assemblages based on clustering with reference sequences representing known taxa obtained from GenBank (shown in red), with accession numbers displayed on the corresponding labels. Scale bars indicate the number of substitutions per site. Species and assemblages identified in this study are highlighted in green or red according to their zoonotic status. The figure also indicates the host species in which each *Giardia* species or *Cryptosporidium* assemblage was detected. (Icons from phylopic.org). Accession numbers for sequences generated by this study: *Cryptosporidium* 18S SSU: GenBank PX970961-PX970982, *Giardia Bg*: GenBank PX986065-PX986077, *Tpi*: GenBank PX986093-PX986100 and *Gdh*: GenBank PX986078-PX986092.

Phylogenetic analyses revealed the presence of multiple *Cryptosporidium* species across the sampled hosts (Fig. 4A). Sample sequences clustered with reference sequences of *C. ubiquitum* (n = 2), *C. ryanae* (n = 3), and *C. andersoni* (n = 6) with strong bootstrap support (≥70%.). A further group of sequences clustered with reference sequences of both *C. bovis* and *C. xiaoi*, which could not be reliably distinguished at this locus; these samples were therefore conservatively classified as *C. bovis/xiaoi* (n = 11).

Of the successful sequences for *Giardia*, most clustered with reference sequences of *G. duodenalis* assemblage E (n = 24) (Fig. 4B). Three sequences clustered within assemblage A, of which two grouped with sub-assemblage AII reference sequences and one with sub-assemblage AI (Fig. 4C and D), supported by bootstrap values ≥ 70%. One sample (K7 goat) was identified as Assemblage AII at the *Tpi* locus but Assemblage E at the GDH locus.

## 4 Discussion

We recorded high levels of *Giardia* and low levels of *Cryptosporidium* occurrence in wildlife and livestock faecal samples in central Laikipia. We detected *Giardia* across all host species, including both assemblages A and E. Despite lower sample prevalence, *Cryptosporidium* exhibited greater species diversity. As only a subset of samples were successfully characterised, further sequencing could help to identify additional rare species and assemblages. Many of the *Giardia* assemblages and *Cryptosporidium* species identified in this study were detected in multiple wildlife and livestock host species. This overlap provides evidence for potential interspecific pathogen sharing likely through contaminated environmental sources such as shared grazing areas or water points (Muloi et al., 2025). Although we identified potentially zoonotic species of *Cryptosporidium* and Assemblages of *Giardia*, their relatively low occurrence indicates that whilst wildlife and livestock could be responsible for human cases they are unlikely to be major sources of human infections.

We recorded widespread evidence of coinfections, suggesting both parasites may share common transmission routes or environmental reservoirs, such as contaminated water sources used by multiple host species. Co-exposure to (oo)cysts in these shared habitats could be one explanation of the elevated coinfection rates. Another possible cause of high coinfections could be that infection with one parasite increases host susceptibility to the other through local immune modulation or gut disturbance (Saleh et al., 2025). Similarly, host factors, including variation in immune status, age, nutritional condition and physiological stress, may influence susceptibility, increasing the likelihood of coinfection with multiple parasites. These findings highlight the need to consider coinfection dynamics when assessing transmission risks at the human–livestock–wildlife interface, further work such as environmental sampling and measuring host immune response would be required to determine the mechanisms behind coinfections.

We positively identified *Giardia* infections in all ten sampled host species. This demonstrates its widespread distribution in wild and domestic herbivores across these landscapes. Of the 10 species, *Giardia* infections have previously been reported in camel (Elmahallawy et al., 2023), cattle (Peter et al., 2015), goat, Grevy's zebra (Mwatenga, 2017) and sheep (Kanyari et al., 2009) in Africa, and in giraffes (Zhao et al., 2025) in captive zoo populations. To our knowledge, *Giardia* infection has not previously been reported in Cape buffalo, plains zebra, African elephant or impala. *Giardia* infections have been reported in forest buffalo (*Syncerus caffer nanus*) in Rwanda which is closely related to the Cape buffalo (Hogan et al., 2014). Similarly, *Cryptosporidium* infections were recorded in 5 of the 10 species. *Cryptosporidium* infections have previously been described in all of these host species: cattle (Kang'ethe et al., 2012), sheep (Essendi et al., 2022), goats (Walter et al., 2021), dromedary camel (Elmahallawy et al., 2023) and impala (Samra et al., 2013).

The absence of significant differences in observed *Giardia* and *Cryptosporidium* prevalence between rainfall periods suggests that infection dynamics in this system may be relatively stable across temporal scales. This pattern could indicate that environmental contamination or host-to-host transmission occurs year-round, independent of seasonal rainfall patterns. Previous studies have detected both higher and lower (oo)cyst concentrations across wet and dry seasons as reviewed in Wang et al. (2023). In this study, *Cryptosporidium* prevalence was lower in samples collected in 2022 during the drought, however whether this was due to seasonal factors or the smaller sample size is hard to determine. However, it is also possible that sampling resolution or sample size limited the power to detect subtle temporal differences.

The dominant assemblage of *G*. *duodenalis* circulating in wildlife and livestock populations in our study areas was assemblage E. This assemblage is widely found across wild and domestic ungulates and has been recorded the most in non-human mammals globally (Hatam-Nahavandi et al., 2025). We found it widely in both domestic (cattle, sheep, goat, dromedary camel) and wild (impala and plains zebra) ungulates. Assemblage E has been regularly reported in cattle (Taghipour et al., 2022), sheep and goats (Li et al., 2025). To our knowledge this is the first time that this assemblage has been reported in either dromedary camel, impala or plains zebra. *Giardia duodenalis* assemblage A was also found in domestic ungulates (cattle and goat). This is an important finding, as assemblage A is a key zoonotic pathogen, and, together with assemblage B, accounts for most human giardiasis worldwide (Hunter and Thompson, 2005). Assemblage A has been widely identified in humans in Kenya (Barasa et al., 2024; Mbae et al., 2016).

Both sub-assemblage AI and AII were identified. Sub-assemblage AI is typically associated with livestock and other animals (e.g. cattle, sheep, goats, dogs, and wildlife) but can occasionally infect humans, indicating zoonotic potential. In contrast, sub-assemblage AII is predominantly human-specific and is the sub-assemblage most frequently reported from human clinical cases worldwide (Sun et al., 2023). Assemblage E is predominantly livestock-adapted (ruminants) and only rarely reported in humans; nonetheless, sporadic human infections have been confirmed (Fantinatti et al., 2016). Humans in these regions are in close contact with their animals, including through shared water sources. The presence of potentially zoonotic pathogens in livestock therefore may create potential for cross-transmission events, however the dominance of animal adapted assemblage E implies that this risk is likely low, and most infections detected in this study are more likely linked with animal associated transmission. One sample was identified as Assemblage AII at the *Tpi* locus but Assemblage E at the *Gdh* locus. This likely means this individual was co-infected with both assemblages.

The detection of four or five species of *Cryptosporidium* in this study shows high diversity on this landscape. Whilst not of as big a concern globally as *C. parvum, C. ubiquitum* is a recognised zoonoses with a broad host range (Ryan et al., 2021). In this study subtyping of *C. ubiquitum* was not possible however Its detection in both impala and sheep in this study suggests the potential for cross-species transmission at the wildlife–livestock interface. *C. ubiquitum* subtype XIIa has previously been reported from a wide range of domestic and wild ungulates, as well as humans (Li et al., 2014), indicating limited host specificity and the capacity to persist in multi-host systems. The presence of this species in sympatric wildlife and livestock therefore highlights its potential to be maintained through shared grazing areas, water sources, or environmental contamination. However, due to the lack of subtyping potential for interspecies transmission can only be inferred. Further work would be required to confirm if the same subtypes were found across our samples and whether they had zoonotic potential. *C. ubiquitum* has previously been identified in impala in Kruger National Park, South Africa (Samra et al., 2013) and has been widely identified in domestic small ruminants (Guo et al., 2021).

We found *C. andersoni* only in cattle which are the primary recorded host species however it has also previously been found in humans (Jiang et al., 2014). *C. ryanae* was found in cattle and one impala. Whilst cattle are the main recorded host of *C. ryanae* (Fayer et al., 2008), to our knowledge this is the first time it has been recorded in impala. In this study we could not distinguish between the two very closely related species *C. xiaoi* and *C. bovis*. These were found in sheep, goats and cattle. Both these species have been identified previously in all these hosts, however *C. bovis* is more commonly reported in cattle (Fayer et al., 2005) and *C. xiaoi* in sheep and goats (Guo et al., 2021). Both species have additionally been reported in humans (Ryan et al., 2021).

Among the five key species examined, plains zebra exhibited the lowest sample prevalence of both *Giardia* and *Cryptosporidium* infections. Phylogenetically, zebras are the most distinct of the key study species, belonging to the family Equidae rather than Bovidae. *Giardia duodenalis* assemblage E was the predominant genotype detected in this study. Although assemblage E has previously been reported in equids (Mizani et al., 2025), it is far more commonly associated with ruminant and swine hosts (Feng and Xiao, 2011). Similarly, the *Cryptosporidium* genotypes identified in this study were predominantly bovid-specific species, which may help explain the lower infection observed in plains zebra. Molecular interpretation is limited as only a small number of zebra samples were successfully sequenced for *Giardia* and none for *Cryptosporidium*, however this evidence may suggest that zebra infections are more likely a result of spillover from other hosts or environmental contamination at shared resources.

Overall the observed *Giardia* sample prevalence across sites exceeded the recently reported global mean for herbivorous mammals (Hatam-Nahavandi et al., 2025). Whilst few studies have been done on these pathogens in non-primate wild mammals in East Africa, previous studies in livestock in other regions of Kenya found lower *Giardia* prevalences in both cattle (Kanyari et al., 2010; Peter et al., 2015), and sheep and goats (Kanyari et al., 2009) compared to our study. Slightly higher prevalences of *Cryptosporidium* were reported in cattle in previous studies in different regions of Kenya (Peter et al., 2015; Ogendo et al., 2017; Kang'ethe et al., 2012; Essendi et al., 2022) when compared to our result. In sheep, the previously reported prevalence of *Cryptosporidium* (Essendi et al., 2022) was significantly higher than our findings. However in goats our recorded sample prevalence was similar to that reported by Essendi et al. (2022). These previous studies were conducted using microscopy methods as their primary means of detection. PCR based detection methods are considered more sensitive and accurate than morphological identification as low level infections are more likely to be detected and there is no chance of misidentification (Morgan et al., 1998; Emisiko et al., 2020). Similarly, differences in host ages matter; with both *Cryptosporidium* and *Giardia,* infections are consistently higher in young animals. Many of the previous *Cryptosporidium* studies in Kenya focus on young livestock due to the higher risk from the disease and the potential for reduced growth rates and survival to adulthood (Peter et al., 2015; Ogendo et al., 2017; Kang'ethe et al., 2012). This could explain the higher prevalences observed in other studies as the majority of our sampling was from adult animals.

We acknowledge that several limitations should be considered when interpreting these findings. Sampling was opportunistic, which limited our ability to account for potential bias around herds, sites, or at sampling events. Although samples were collected during periods corresponding to different rainfall conditions, these periods were coarse and sample sizes within some species–year combinations were small, reducing power to detect subtle temporal effects. In addition, the absence of detailed host- and environment-level covariates precluded formal risk-factor modelling, and prevalence estimates should therefore be interpreted descriptive patterns of sample level infection rather than evidence of epidemiological drivers. Measures were taken to avoid repeat sampling and to our best knowledge were successful, however due to the nature of the landscape we cannot completely guarantee that none occurred. The PCR methods used are sensitive but can only determine whether an individual was positive or negative for the parasites and could not be used to quantify infection intensity. Finally, genotyping was only successful for a subset of positive *Giardia* samples, limiting the resolution of assemblage identification across species. Similarly, *C. ubiquitum* subtyping and distinguishing between *C. bovis* and *C. Xiaoi* requires further molecular work such as *gp60* analysis, this would provide more insight into the potential of inter-species transmission and potential zoonoses. Future work incorporating finer-scale longitudinal sampling, environmental surveillance, and expanded molecular characterisation across humans, livestock, wildlife, and shared resources would help to better resolve transmission dynamics at this interface.

The detection of potentially zoonotic genotypes (e.g. *G*. *duodenalis* assemblage A and *C*. *ubiquitum*) alongside livestock-adapted genotypes of *Giardia* and *Cryptosporidium* highlights possible One Health implications of these pathogens across the landscape studied. Some subtypes of these pathogens are recognised causes of diarrhoea in humans, particularly children and immunocompromised individuals (Prabakaran et al., 2023). Their detection in livestock and, in the case of *C. ubiquitum*, wildlife, indicates a possible shared exposure and potential public health risk through transmission from animals to humans. However, it should be noted that we cannot demonstrate transmission pathways, and the low occurrence of these potentially zoonotic strains likely implies that the risk of zoonotic transmission, if present, is low. In contrast, more ungulate-specific genotypes such as *G. duodenalis* assemblage E and *C. andersoni*, were detected more frequently and are generally considered to have limited zoonotic potential (Feng and Xiao, 2011; Bulumulla et al., 2025). Their presence across wildlife and livestock therefore suggests that animal-associated transmission cycles may predominate, rather than direct animal to human zoonotic risk, although indirect effects on public health through livestock productivity and growth (Abeywardena et al., 2015) remain important. Infection with these pathogens may also have potential impacts on wildlife condition and survival, although their effects on wild animals remain debated (Appelbee et al., 2005).

Improved molecular screening across humans, livestock, wildlife and environmental reservoirs would allow enhanced understanding of infection sources and transmission pathways that cannot be determined by this study, such as determining whether interspecies and zoonotic transmission is actually occurring on this landscape. This information would enable more effective management of diarrhoeal disease around human/wildlife/livestock interfaces and could assist in informing practical measures such as identifying transmission hotspots, separating shared waterpoints, and facilitating behavioural changes and changes to livestock management. This could help reduce risk and exposure to these pathogens and mitigate health and economic impacts across communities and ecosystems. Many pastoralist communities are already advocating for changes such as water sources that separate where humans, livestock and wildlife drink. However, without access to public support for better water infrastructure there is currently no choice but to share potentially dangerous water sources especially during periods of water scarcity.

## Funding

This work was supported by an 10.13039/501100000266EPSRC studentship awarded to Andrew Halls (grant number 2865560), a 10.13039/501100000268BBSRC studentship awarded to Alice Burton (grant number 2594562), and funding awarded to Susanne Shultz from the 10.13039/501100000770University of Manchester Research Institute (2023–2024), the International Science Partnership Fund (2024–2025), the 10.13039/501100023278Darwin Initiative (grant number DARNV025), and 10.13039/501100000288The Royal Society (grant number UF110641).

## CRediT authorship contribution statement

**Andrew M. Halls:** Conceptualization, Data curation, Formal analysis, Investigation, Methodology, Project administration, Validation, Visualization, Writing – original draft, Writing – review & editing. **Alice M. Burton:** Investigation, Writing – review & editing. **Olatunji Johnson:** Conceptualization, Funding acquisition, Methodology, Supervision, Writing – review & editing. **Catherine Walton:** Funding acquisition, Methodology, Supervision, Writing – review & editing. **Charis Enns:** Funding acquisition, Supervision, Writing – review & editing. **Kathryn J. Else:** Funding acquisition, Supervision, Writing – review & editing. **Ian Hall:** Methodology, Supervision, Writing – review & editing. **Melanie Wangui:** Writing – review & editing. **Cianjoka Gichuyia:** Writing – review & editing. **Eric M. Fèvre:** Funding acquisition, Writing – review & editing. **Susanne Shultz:** Conceptualization, Funding acquisition, Methodology, Project administration, Supervision, Writing – review & editing.

## Declaration of competing interest

The authors declare that they have no known competing financial interests or personal relationships that could have appeared to influence the work reported in this paper.

## Footnotes

Appendix A Supplementary data to this article can be found online at https://doi.org/10.1016/j.ijppaw.2026.101263.
