# Supplementary material for: Prevalence and molecular characterisation of Giardia spp. and Cryptosporidium spp. at the wildlife-livestock interface in Laikipia, Kenya
Source: Int J Parasitol Parasites Wildl. 2026 Jul 20;30:101263. doi: 10.1016/j.ijppaw.2026.101263 (PMC13416461; doi:10.1016/j.ijppaw.2026.101263)
Supplement: Multimedia component 1 [file mmc1.docx]

Appendices

| Target | Reaction | Forward Primer | Reverse Primer | Expected product | Reference |
| --- | --- | --- | --- | --- | --- |
| Giardia 18S SSU | Primary | 5′-CATCCGGTCGATCCTGCC-3′ | 5′-AGTCGAACCCTGATTCTCCGCCAGG-3′ | 292bp | (ElBakri et al., 2014) |
|  | Nested | 5′-GACGCTCTCCCCAAGGAC-3′ | 5′- CTGCGTCACGCTGCTCG -3′ | 130bp |  |
| Cryptosporidium 18S SSU | Primary | 5′-TTCTAGAGCTAATACATGCG-3′ | 5′- CCCTAATCCTTCGAAACAGGA-3′ | 1,325 bp | (Xiao et al., 1999) |
|  | Nested | 5′- GGAAGGGTTGTATTTATTAGATAAAG-3 | 5′- AAGGAGTAAGGAACAACCTCCA-3′ | 826 to 864bp |  |
| Giardia Bg | Primary | 5’-AAGCCCGACGACCTCACCCGCAGTGC-3’ | 5’-GAGGCCGCCCTGGATCTTCGAGACGAC-3’ |  | (Kuthyar et al., 2021) |
|  | Nested | 5’- GAACGAACGAGATCGAGGTCCG-3’ | 5’-CTCGACGAGCTTCGTGTT-3’ | 511bp |  |
| Giardia Tpi | Primary | 5’-AAATIATGCCTGCTCGTCG-3’ | 5’-CAAACCTTITCCGCAAACC-3’ |  | (Sulaiman et al., 2003) |
|  | Nested | 5’-CCCTTCATCGGIGGTAACTT-3’ | 5’-GTGGCCACCACICCCGTGCC-3’ | 530bp |  |
| Giardia Gdh | Primary | 5′-TCAACGTYAAYCGYGGYTTCCGT-3′ | 5′-GTTRTCCTTGCACATCTCC-3′ |  | (Read et al., 2004) |
|  | Nested | 5′-CAGTACAACTCYGCTCTCGG-3′ |  | 432bp |  |

Table A1 – Primers used for PCR

Table A2 – Reaction conditions for PCRS

| Target | Reaction | Initial denaturing | | Denaturing | | Annealing | | Extension | | Final extension | | Cycles | Reference |
| --- | --- | --- | --- | --- | --- | --- | --- | --- | --- | --- | --- | --- | --- |
|  |  | Time | Temp (˚C) | Time | Temp (˚C) | Time | Temp (˚C) | Time | Temp (˚C) | Time | Temp (˚C) |  |  |
| Giardia 18S SSU | Primary & Nested | 4m | 96 | 20s | 96 | 20s | 59 | 30s | 72 | 7m | 72 | 35 | (ElBakri et al., 2014) |
| Cryptosporidium 18S SSU | Primary & Nested | 3m | 94 | 45s | 94 | 45s | 56 | 1m | 72 | 7m | 72 | 35 | (Xiao et al., 1999) |
| Giardia Bg | Primary | 3m | 95 | 20s | 95 | 30s | 65 | 1m | 72 | 10m | 72 | 35 | (Kuthyar et al., 2021) |
|  | Nested | 3m | 95 | 20s | 95 | 30s | 64 | 1m | 72 | 10m | 72 | 35 |  |
| Giardia Tpi | Primary & Nested | 5m | 94 | 45s | 94 | 45s | 50 | 1m | 72 | 10m | 72 | 35 | (Sulaiman et al., 2003) |
| Giardia Gdh | Primary & Nested | 3m | 95 | 30s | 95 | 30s | 55 | 1m | 72 | 7m | 72 | 35 | (Read et al., 2004) |


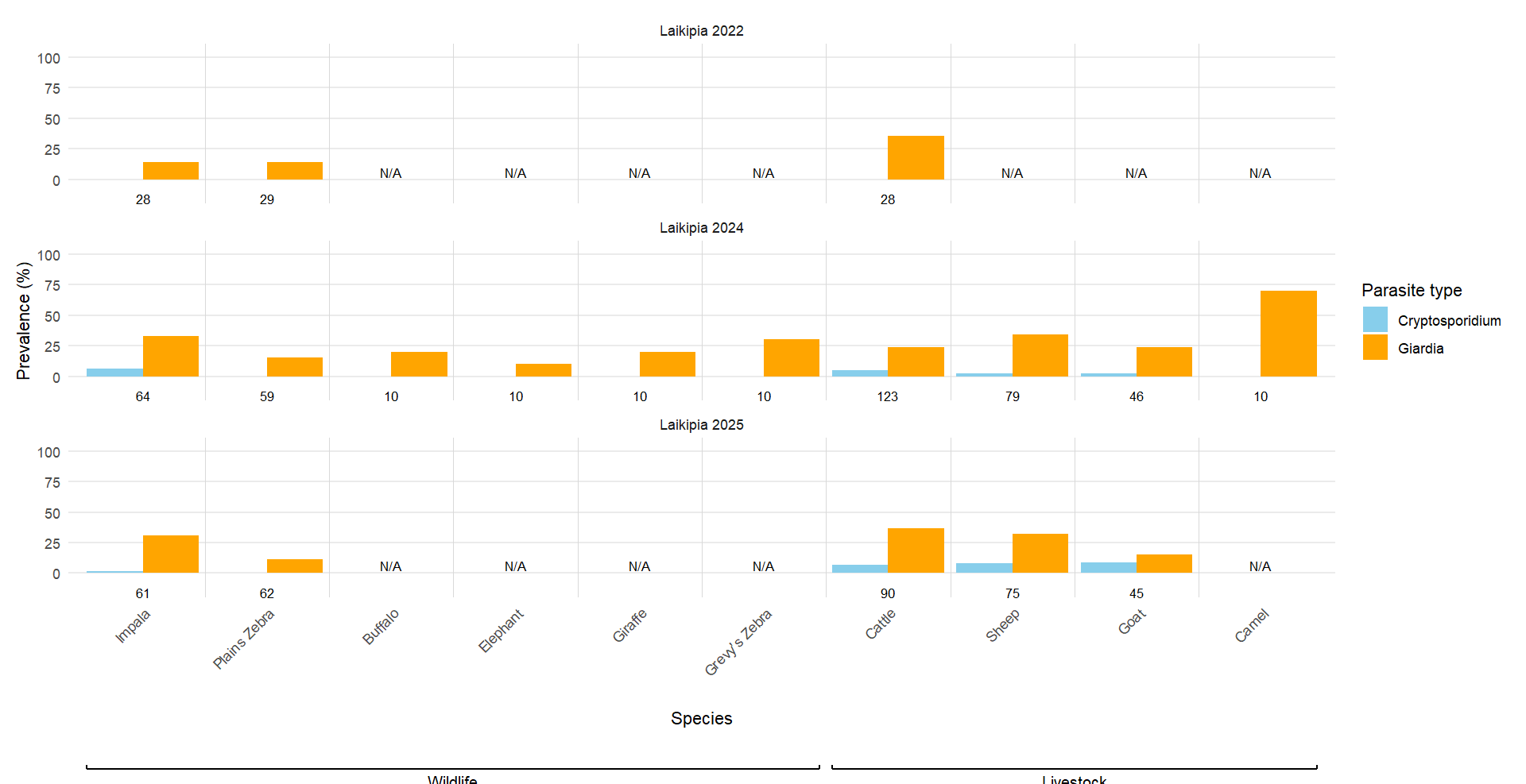


Figure A1 - Prevalence of Giardia and Cryptosporidium across ungulate species and datasets.
Bar plots show the observed prevalence (%) of Giardia (orange) and Cryptosporidium (blue) in domestic and wild ungulates sampled in Laikipia (2022, 2024, 2025). Each panel represents a year. Numbers under the bars indicate the total number of individuals sampled per species; “N/A” indicates that a species was not sampled in that dataset. Prevalences are raw proportions of positive individuals,

Table A3 - Model coefficients from binomial generalised linear models (GLMs) examining predictors of Giardia infection, Cryptosporidium infection, and combined protozoan infection likelihood across host species, counties, and sampling years. The table reports parameter estimates (log-odds scale), standard errors (SE), z-values, and associated p-values for each model term. For the Giardia-only and Cryptosporidium-only models, the response variable was infection status for the corresponding parasite. For the combined model, infection status was modelled with parasite species (Giardia vs. Cryptosporidium) included as an additional predictor. Reference levels were Cattle (Species), 2022 (Year), and Giardia (Parasite; combined model only). Negative coefficients indicate lower infection odds relative to the reference category, whereas positive coefficients indicate higher odds. Very large standard errors and unstable estimates in the Cryptosporidium model reflect sparse infection data. All models were fitted using a binomial error distribution with a logit link. Bold indicates significant.

| Term | Estimate | SE | z_value | p_value |
| --- | --- | --- | --- | --- |
| *Giardia* | | | | |
| (Intercept) | **-1.02** | **0.30** | **-3.44** | **< 0.001** |
| Year2024 | 0.15 | 0.30 | 0.50 | 0.62 |
| Year2025 | 0.23 | 0.31 | 0.74 | 0.46 |
| Species: Goat | -0.57 | 0.30 | -1.91 | 0.06 |
| Species: Impala | -0.05 | 0.23 | -0.20 | 0.84 |
| Species: Plains Zebra | **-1.01** | **0.28** | **-3.62** | **< 0.001** |
| Species: Sheep | 0.12 | 0.22 | 0.56 | 0.58 |
| *Cryptosporidium* | | | | |
| (Intercept) | -4.54 | 1.40 | -3.24 | < 0.01 |
| Year2024 | 1.57 | 1.42 | 1.11 | 0.27 |
| Year2025 | 2.00 | 1.42 | 1.41 | 0.16 |
| Species: Goat | -0.01 | 0.53 | -0.01 | 0.99 |
| Species: Impala | -0.34 | 0.52 | -0.65 | 0.51 |
| Species: Plains Zebra | **-2.75** | **1.42** | **-1.94** | **0.05** |
| Species: Sheep | -0.09 | 0.46 | -0.21 | 0.84 |
| Combined | | | | |
| (Intercept) | **-1.13** | **0.29** | **-3.95** | **< 0.001** |
| Year: 2024 | 0.27 | 0.29 | 0.93 | 0.35 |
| Year: 2025 | 0.41 | 0.29 | 1.40 | 0.16 |
| Parasite: Crypto | **-2.22** | **0.20** | **-10.85** | **< 0.001** |
| Species: Goat | -0.47 | 0.27 | -1.74 | 0.08 |
| Species: Impala | -0.10 | 0.21 | -0.48 | 0.63 |
| Species: Plains Zebra | **-1.16** | **0.27** | **-4.30** | **< 0.001** |
| Species: Sheep | 0.09 | 0.20 | 0.43 | 0.67 |

Table A4 - Estimated marginal means (EMMeans) for predicted infection probabilities from binomial generalised linear models (GLMs) examining Giardia, Cryptosporidium and combined protozoan infection patterns across host species and sampling years. The table provides model-adjusted predicted probabilities (prob) on the response scale, together with standard errors (SE), degrees of freedom (df), and 95% confidence intervals (CI_low–CI_high). For parasite-specific models, estimates are averaged over Year; for the combined model, estimates are averaged over parasite species or host species as appropriate. Extremely broad confidence intervals for Cryptosporidium reflect the very low number of positive detections, which leads to unstable probability estimates near zero.

| Factor | Prob (%) | SE  (%) | df | CI_low  (%) | CI_high (%) |
| --- | --- | --- | --- | --- | --- |
| *Giardia* | | | | | |
| Species: Cattle | 29.11 | 3.21 | Inf | 23.23 | 35.77 |
| Species: Goat | 18.80 | 4.28 | Inf | 11.78 | 28.63 |
| Species: Impala | 28.16 | 3.74 | Inf | 21.44 | 36.03 |
| Species: Plains Zebra | 12.99 | 2.77 | Inf | 8.46 | 19.44 |
| Species: Sheep | 31.75 | 4.27 | Inf | 24.02 | 40.62 |
| *Cryptosporidium* | | | | | |
| Species: Cattle | 3.39 | 1.71 | Inf | 1.25 | 8.88 |
| Species: Goat | 3.37 | 2.10 | Inf | 0.98 | 10.96 |
| Species: Impala | 2.44 | 1.42 | Inf | 0.77 | 7.46 |
| Species: Plains Zebra | 0.22 | 0.32 | Inf | 0.01 | 3.68 |
| Species: Sheep | 3.09 | 1.76 | Inf | 1.00 | 9.17 |
| Combined | | | | | |
| Species: Cattle | 11.83 | 1.66 | Inf | 8.94 | 15.49 |
| Species: Goat | 7.77 | 1.88 | Inf | 4.80 | 12.34 |
| Species: Impala | 10.81 | 1.79 | Inf | 7.77 | 14.86 |
| Species: Plains Zebra | 4.03 | 0.99 | Inf | 2.49 | 6.47 |
| Species: Sheep | 12.77 | 2.15 | Inf | 9.11 | 17.61 |
| Parasite: Giardia | 22.65 | 2.00 | Inf | 18.97 | 26.82 |
| Parasite: Crypto | 3.09 | 0.62 | Inf | 2.09 | 4.55 |

Table A5 - Pairwise contrasts of estimated marginal means (EMMeans) from binomial generalised linear models (GLMs) examining Giardia, Cryptosporidium and combined protozoan infection across host species and sampling years. The table reports log-odds contrasts between factor levels, expressed as estimated differences (OR), standard errors (SE), 95% confidence intervals (CI_low–CI_high), and associated p-values. For parasite-specific models, contrasts are averaged over Year; for the combined model, contrasts are averaged across the relevant remaining predictors. Contrasts with wide confidence intervals, particularly for Cryptosporidium, reflect the very low number of positive detections, which results in unstable parameter estimates and reduced precision. Bold indicates significant.

| Contrast | OR | SE | CI_low | CI_high | df | p.value |
| --- | --- | --- | --- | --- | --- | --- |
| *Giardia* | | | | | | |
| Cattle/Goat | 0.57 | 0.30 | -0.25 | 1.39 | Inf | 0.31 |
| Cattle/Impala | 0.05 | 0.23 | -0.58 | 0.67 | Inf | 1.00 |
| Cattle/Plains Zebra | **1.01** | **0.28** | **0.25** | **1.77** | **Inf** | **< 0.01** |
| Cattle/Sheep | -0.12 | 0.22 | -0.74 | 0.49 | Inf | 0.98 |
| Goat/Impala | -0.53 | 0.32 | -1.41 | 0.35 | Inf | 0.47 |
| Goat/Plains Zebra | 0.44 | 0.36 | -0.54 | 1.42 | Inf | 0.74 |
| Goat/Sheep | -0.70 | 0.31 | -1.55 | 0.16 | Inf | 0.17 |
| Impala/Plains Zebra | **0.97** | **0.30** | **0.15** | **1.78** | **Inf** | **0.01** |
| Impala/Sheep | -0.17 | 0.25 | -0.86 | 0.52 | Inf | 0.96 |
| Plains Zebra/Sheep | **-1.14** | **0.30** | **-1.95** | **-0.32** | **Inf** | **< 0.01** |
| *Cryptosporidium* | | | | | | |
| Cattle/Goat | 0.01 | 0.53 | -1.43 | 1.44 | Inf | 1.00 |
| Cattle/Impala | 0.34 | 0.52 | -1.07 | 1.74 | Inf | 0.97 |
| Cattle/Plains Zebra | 2.75 | 1.42 | -1.11 | 6.62 | Inf | 0.29 |
| Cattle/Sheep | 0.09 | 0.46 | -1.15 | 1.34 | Inf | 1.00 |
| Goat/Impala | 0.33 | 0.62 | -1.35 | 2.01 | Inf | 0.98 |
| Goat/Plains Zebra | 2.75 | 1.46 | -1.22 | 6.72 | Inf | 0.32 |
| Goat/Sheep | 0.09 | 0.56 | -1.45 | 1.63 | Inf | 1.00 |
| Impala/Plains Zebra | 2.42 | 1.45 | -1.54 | 6.37 | Inf | 0.46 |
| Impala/Sheep | -0.24 | 0.56 | -1.76 | 1.27 | Inf | 0.99 |
| Plains Zebra/Sheep | -2.66 | 1.43 | -6.57 | 1.25 | Inf | 0.34 |
| Combined | | | | | | |
| Cattle/Goat | 0.47 | 0.27 | -0.26 | 1.20 | Inf | 0.41 |
| Cattle/Impala | 0.10 | 0.21 | -0.47 | 0.67 | Inf | 0.99 |
| Cattle/Plains Zebra | **1.16** | **0.27** | **0.42** | **1.90** | **Inf** | **< 0.001** |
| Cattle/Sheep | -0.09 | 0.20 | -0.64 | 0.47 | Inf | 0.99 |
| Goat/Impala | -0.36 | 0.29 | -1.15 | 0.42 | Inf | 0.72 |
| Goat/Plains Zebra | 0.70 | 0.34 | -0.22 | 1.61 | Inf | 0.23 |
| Goat/Sheep | -0.55 | 0.28 | -1.32 | 0.21 | Inf | 0.28 |
| Impala/Plains Zebra | **1.06** | **0.29** | **0.27** | **1.85** | **Inf** | **< 0.01** |
| Impala/Sheep | -0.19 | 0.23 | -0.82 | 0.44 | Inf | 0.93 |
| Plains Zebra/Sheep | **-1.25** | **0.29** | **-2.03** | **-0.47** | **Inf** | **< 0.001** |
| Giardia/Crypto | **2.22** | **0.20** | **1.82** | **2.62** | **Inf** | **< 0.0001** |

Table A6 – Robustness analysis of generalised linear mixed models (GLMMS) with year as a random intercept. Fixed effects were assessed using Type III Wald χ² test from binomial GLMMs with a logit link. Between year variability was assessed using the estimated variance and standard deviation (SD). For all models the variance associated with year collapsed to zero indicating no evidence of differences in infection prevalence between years. For Cryptosporidium, low infection prevalence reduced statistical power in the random-effects model, leading to weaker species effects; however, effect directions were consistent with the fixed-effect analysis and overall conclusions were unchanged.

| Model | Effect | χ² | Df | p-value | Year variance | Year SD |
| --- | --- | --- | --- | --- | --- | --- |
| Giardia | Species | **19.85** | **4** | **< 0.001** | 0.00 | 0.00 |
| Cryptosporidium | Species | 0.94 | 4 | 0.92 | 0.00 | 0.00 |
| Combined (both parasites) | Species | **24.63** | **4** | **< 0.001** | 0.00 | 0.00 |
|  | Parasite | **117.66** | **1** | **< 0.001** | 0.00 | 0.00 |
